# A Brazilian series utilizing the SMASH-U system for etiologic classification of intracerebral hemorrhage

**DOI:** 10.1055/s-0044-1779505

**Published:** 2024-02-05

**Authors:** Luiz Henrique Libardi Silva, João Brainer Clares de Andrade, Ahmad Ali El Majdoub, Maramélia Miranda-Alves, Raul Alberto Valiente, Daniela Laranja Gomes Rodrigues, Gisele Sampaio Silva

**Affiliations:** 1 Universidade Federal de São Paulo, São Paulo SP, Brazil.; 2 Centro Universitário São Camilo, São Paulo SP, Brazil.; 3 Hospital Israelita Albert Einstein, São Paulo SP, Brazil.

**Keywords:** Hemorrhagic Stroke, Prognosis, Outcome Assessment, Health Care, Acidente Vascular Cerebral Hemorrágico, Prognóstico, Avaliação de Resultados em Cuidados de Saúde

## Abstract

**Background** Understanding the causes of intracerebral hemorrhage (ICH) is crucial for effective treatment and preventing recurrences. The SMASH-U scale is a suggested method for classifying and predicting the outcomes of ICH.

**Objective** To describe the SMASH-U classification and outcomes by etiology in patients admitted to a comprehensive stroke center in São Paulo, Brazil.

**Methods** A retrospective analysis was conducted on patients admitted to the hospital or outpatient clinic between April 2015 and January 2018. Two stroke neurologists evaluated the SMASH-U classification, and patients with incomplete medical records were excluded.

**Results** Out of the 2000 patients with a stroke diagnosis evaluated, 140 were included in the final analysis. The mean age was 57.9 (± 15.5) years, and 54.3% were male. Hypertension was the most frequent etiology, accounting for 41.4% of cases, followed by amyloid angiopathy (18.5%) and structural lesions (14.1%). Structural lesions were more common among women and patients under 45 years old. Favorable outcomes were observed in 61% of patients with structural lesions, compared to 10% of patients with medication-related etiologies.

**Conclusion** This study provides important evidence regarding the etiological classification of Brazilian patients with ICH. Hypertension and amyloid angiopathy were the most frequent causes, while structural lesions and systemic diseases were more common in younger patients.

## Resumo

**Antecedentes** Compreender as causas da hemorragia intracerebral (HIC) é crucial para o tratamento eficaz e prevenção de recorrências. A escala SMASH-U é um método sugerido para classificar e prever os resultados da HIC.

**Objetivo** Descrever a classificação SMASH-U e os resultados por etiologia em pacientes admitidos em um centro de acidente vascular cerebral (AVC) em São Paulo, Brasil.

**Métodos** Foi realizada uma análise retrospectiva de pacientes admitidos no hospital ou ambulatório entre abril de 2015 e janeiro de 2018. Dois neurologistas especializados em doenças cerebrovasculares avaliaram a classificação SMASH-U e pacientes com prontuários incompletos foram excluídos.

**Resultados** Dos 2000 pacientes com diagnóstico de AVC avaliados, 140 foram incluídos na análise final. A idade média foi de 57,9 (±15,5) anos e 54,3% eram do sexo masculino. A hipertensão foi a etiologia mais frequente, correspondendo a 41,4% dos casos, seguida pela angiopatia amiloide (18,5%) e lesões estruturais (14,1%). As lesões estruturais foram mais comuns em mulheres e pacientes com menos de 45 anos. Resultados favoráveis foram observados em 61% dos pacientes com lesões estruturais, em comparação com 10% dos pacientes com etiologias relacionadas a medicamentos.

**Conclusão** Este estudo fornece evidências importantes sobre a classificação etiológica de pacientes brasileiros com HIC. A hipertensão e a angiopatia amiloide foram as causas mais frequentes, enquanto lesões estruturais e doenças sistêmicas foram mais comuns em pacientes mais jovens.

## INTRODUCTION

Intracerebral hemorrhage (ICH) is responsible for 10-20% of cerebrovascular events. ICH can occur due to the rupture of small blood vessels, which is often attributed to conditions such as systemic arterial hypertension and amyloid angiopathy, or due to abnormalities in blood vessels such as arteriovenous malformations (AVMs), aneurysms, arteriovenous fistulas, and cavernomas. 1 2 3

**Figure 1:**
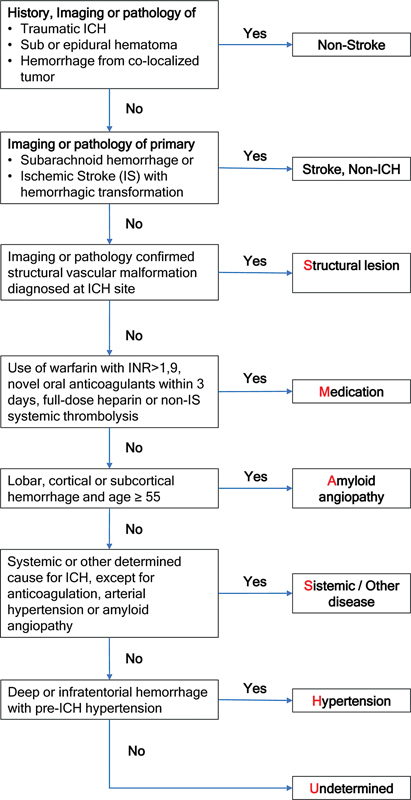
Notes: *Hematoma volume on admission head Computerized Tomography Scan, calculated using the ABC/2 method. 4 (Adapted from Merejota 4 ). The SMASH-U classification. Abbreviation: ICH, Intracrebral Hemorrhage.

In 2012, a new classification system for ICH was proposed, called SMASH-U, 4 which categorizes ICH based on various etiologies such as structural lesions (SL), medication-related lesions (M), amyloid angiopathy (A), systemic disease (S), hypertension (H), and undetermined (U) etiologies ( Figure 1 ). In most parts of the world, systemic arterial hypertension and amyloid angiopathy are the leading causes of ICH. However, there have been no studies in Brazil that have systematically examined the etiological classification of ICH. Moreover, there is limited research on the role of the SMASH-U classification in prognostication. 5 6

This study aims to describe the SMASH-U classification and outcomes by etiology in patients followed in a Comprehensive Stroke Center (CSC) located in a public hospital in São Paulo, Brazil.

## METHODS

We carried out a study on epidemiology that looked at past medical records of patients who were admitted to a Comprehensive Stroke Center in Brazil. The study included patients who were admitted between January 1, 2015, and April 30, 2018, and diagnosed with ICH. This included patients who were treated as outpatients at our clinic as well as those admitted to the hospital during the same period. Patients who passed away within the initial 24 hours of hospital admission were excluded from this analysis due to the inability to collect data necessary for the etiological classification of the intracranial hemorrhage.

### Patient selection

We included patients aged over 18 years, of either sex, with a definitive diagnosis of ICH evaluated with computed tomography or magnetic resonance imaging. We excluded patients with ICH resulting from trauma, hemorrhagic transformations of ischemic strokes, primary subarachnoid hemorrhages, and primary subdural or extradural hematomas.

### Data collection and statistical analysis

The following data were collected: age, sex, ethnicity, educational background, medical history of hypertension, diabetes, smoking habits, total cholesterol and LDL levels, alcohol consumption, prior usage of antiplatelet agents, anticoagulants, statins, documented cases of atrial fibrillation (AF), carotid artery stenosis, ischemic stroke, transient ischemic attack (TIA), hemorrhagic stroke, SMASH-U classification, and ICH score 7 . Furthermore, the location of the hematoma (lobar, basal ganglia, thalamus, brainstem, or cerebellum), 8 the presence of intraventricular and infratentorial blood, the hematoma volume (calculated using the formula ABC/2), 7 Glasgow Coma Scale at hospital admission, extension to the subarachnoid space, and surgical evacuation of the hematoma were also recorded. Risk factors were deemed present when documented in the medical record. Images and reports were thoroughly reviewed utilizing an electronic medical image viewer (Synapse® 3D).

The ICH score 7 and the NIHSS 9 stroke scales were assessed using data from medical records upon hospital admission. The modified Rankin Scale was evaluated at the time of patient discharge, following the description provided in the medical record's neurological examination. 10 The SMASH-U classification was independently scored by two investigators trained in the scale's utilization. The flowchart depicted in Figure 1 demonstrates the inclusion criteria considered for each item on the scale. Hypertension was identified as present when documented in medical records or using antihypertensive medication. Amyloid angiopathy was determined using the Boston criteria. 11 12 Discrepant cases were evaluated by a third examiner with 15 years of experience in vascular neurology.

### Statistical analysis

The normality of the tested samples was evaluated using the Shapiro-Wilk test. Categorical variables were presented as frequencies and percentages, while continuous variables were expressed as means and standard deviations. Categorical variables were compared using either the chi-square test or Fisher's exact test. The independent samples t-test was employed for inter-group mean comparisons. Qualitative non-parametric variables underwent assessment via the Mann-Whitney test (a non-parametric test for comparing means or medians), followed by the Bonferroni posthoc test. Given a sample size of 140 patients, the Bonferroni correction was implemented to counteract inflated Type I error rates arising from multiple simultaneous statistical tests. This correction entails adjusting the significance level (p-value) by dividing it by the number of conducted tests. Assuming a common significance level of 0.05 and, for instance, conducting 10 tests, the adjusted significance threshold becomes 0.005. This safeguards a more stringent criterion for asserting statistical significance and effectively governs the overall likelihood of Type I errors across all tests. These analyses were conducted using the SPSS 24.0 software, with a significance level of 5% considered. 13

### Standard protocol approvals, registrations, patient consent, and data availability

The hospital IRB approved this study. All national ethical requirements were met to support this research. The IRB waived the need for informed consent for retrospective data collection. Anonymized data, study protocol, statistical analysis plan, and informed consent form will be shared upon request from other investigators after ethics approval. All data used in the analyses are presented in tables. The number of ethics committee approval is 0393.0070.04/2018.

## RESULTS

A total of 2000 medical records were evaluated, and 140 patients were included in the final analysis. The mean age of the patients was 57.9 years +/- 15.53 years, 54% males. Hypertension was the most frequent risk factor (76.1%), followed by smoking (31.6% prior, 16.3% current), alcohol use, diabetes (21.2%), atrial fibrillation (9.4%), use of antiplatelets (25.2%), and anticoagulants (9.3%) ( Table 1 and 2 ). A total of 8.8% of the patients had coronary artery disease and 11.4% had a history of stroke. The most frequent etiology was hypertension 41.42% (H), followed by amyloid angiopathy 18.57% (A), 11.45% structural lesions (SL), systemic diseases 11.45% (S2), undetermined diseases (U) 10% and medication use 7.1% (M).

**Table 1:** Baseline clinical characteristics (N = 140)

Characteristics		n (%)
Age	Mean (SD)	58.0 (15.5)
	Lower – Higher (n)	17.0–92.7 (140)
Age (dichotomized)	< 45 y	29 (20.7%)
	≥ 45 y	111 (79.3%)
	Total	140 (100.0%)
Gender	Female	64 (45.7%)
	Male	76 (54.3%)
	Total	140 (100.0%)
mRs	0	14 (10.0%)
	1	21 (15.0%)
	2	22 (15.7%)
	3	20 (14.3%)
	4	18 (12.9%)
	5	13 (9.3%)
	6	32 (22.9%)
	Total	140 (100.0%)
mRs 0-2	No	83 (59.3%)
	Yes	57 (40.7%)
	Total	140 (100.0%)
ICH	0	5 (9.4%)
	1	18 (34.0%)
	2	14 (26.4%)
	3	13 (24.5%)
	4	3 (5.7%)
	Total	53 (100.0%)

Abbreviations: ICH, Intracerebral Hemorrhage; mRS, Modifed Rankin Scale; NIH, National Institute of Health.

**Table 2:** Baseline image, laboratory, and risk factors characteristics (N = 140)

Characteristics		n (%)
Initial NIH Stroke Scale	Median [Interquartile range]	13 [6. 18]
	Lower – Higher (n)	0- 24 (43)
ICH site	Lobar	67 (51.5%)
	Deep	56 (43.4%)
	Infratentorial	9 (7%)
	Total	129 (100%)
Intraventricular hemorrhage		43 (33.3%)
INR	Median [Interquartile range]	1.1 [1. 1.2]
	Lower – Higher (n)	0.9 - 9 (111)
Platelets (/mm ^3^ )	Median [Interquartile range]	204000 [150000. 255000]
	Lower – Higher (n)	110 - 537000 (117)
Previous stroke		13 (11.4%)
Atrial fibrillation		10 (9.4%)
Peripheral artery disease		2 (2.0%)
Coronary artery disease		9 (8.8%)
Diabetes mellitus		25 (21.2%)
History of arterial hypertension		89 (76.1%)
Smoking	Never smoke	51 (52.0%)
	Previous smoke	31 (31.6%)
	Smoker	16 (16.3%)
	Total	98 (100.0%)
Alcohol intake	Never drink	56 (62.2%)
	Former alcoholic	16 (17.8%)
	Alcoholic	18 (20.0%)
	Total	90 (100.0%)
Previous antiplatelets use		27 (25.2%)
Previous anticoagulants use		13 (9.3%)
Previous Statins use		36 (34.0%)

Abbreviations: ICH, Intracerebral Hemorrhage; INR, International Normalized Ratio; NIH, National Institute of Health.

Information on the ICH score at admission was available for 53 cases ( Table 3 ), with the most frequent categories being 1 (34.0%), 2 (26.4%), and 3 (24.5%). The initial NIHSS score was obtainable for 43 cases, ranging from 0 to 24, with a median of 13 (interquartile ranges 25, 75: 6; 18.0). In total, 51.5% of the cases exhibited lobar hematomas, 43.4% demonstrated deep supratentorial hematomas, and 7% displayed infratentorial hematomas (5.7% of the patients had more than one topography). Intraventricular hemorrhage was observed in 33% of the patients. The hospital mortality rate was 22.9%, with 40.7% of the patients having a modified Rankin Scale score of 0-2 at hospital discharge. In 77.9% of the evaluations, the two evaluators agreed upon the SMASH-U classification.

**Table 3:** Clinical, laboratory and neuroimaging characteristics by SMASH-U groups

		S1, n(%)	M, n(%)	A, n(%)	S2, n(%)	H, n(%)	U, n(%)	Total, n(%)	p-value 1
Age*	<45 years	10(34.48%)	0(0.0%)	0(10.0%)	7(24.14%)	7(24.14%)	5(17.24%)	29(100%)	<0.001
	≥45 years	6(5.4%)	10(9%)	26(23.92)	9(8.12)	51(45.94%)	9(8.12)	111(100%)	
Male*		5(6.57%)	3(3.95%)	14(18.4%)	7(9.2%)	40(52.68%)	7(9.2%)	76	0.038
mRs	3-6*	5(6%)	9 (10.8%)%)	15(18.1)	8(9.65%)	38(45.8%)	8(9.65%)	83(100%)	0.057
	0-2	11 (19.3%)	1(1.7%)	11(19.3%)	8(14%)	20(35.1%)	6 (10.6%)	57(100%)	0.057
ICH Site – Lobar*		10(12.04%)	6(7.2%)	25(30.12%)	10(12.04%)	7(8.48%)	25(30.12%)	83 (100%)	0.001
ICH Site Infratentorial*		3(33.33%)	0(0%)	1(11.1%)	0(0%)	4(44.47%)	1(11.1%)	9(100%)	0.396
Intraventricular hemorrhage*		0(0%)	2(3.6)	9(17%)	1(1.8%)	28(53.1%)	13(24.5%)	53(100%)	<0.001
ICH Site – Deep / subcortical*		2(3.6%)	1(1.8%)	1(1.8%)	2(3.6%)	46(82%)	4(7.2%)	56(100%)	<0.001
History of Diabetes Mellitus*		1(4%)	4(16%)	7(28%)	1(4%)	8(32%)	4(16%)	25(100%)	0.086
History of Arterial Hypertension*		7(7.5%)	7(7.5)	16(17.2%)	4(4.3%)	53(57.1%)	6(6.4%)	93(100%)	<0.001

Abbreviations: ICH, Intracerebral hemorrhage; mRs, Modified Rankin Scale.

Note: *Chi Square test used.

Among younger patients, the most frequent cause of ICH ( Table 3 ) was structural injury (34.4%), followed by systemic diseases (24.1%) and hypertension (24.1%). For older patients, hypertension (45.9%) and amyloid angiopathy (23.4%) were the main etiologies (p < 0.01). Women had hypertension (28.12%), amyloid angiopathy (18.7%), and structural injury (17.1%) as the main causes, while men had hypertension (52.68%), amyloid angiopathy (18.4%) and systemic diseases and undetermined causes (9.2% each) as the leading etiologies (p = 0.04) ( Table 2 ).

Patients with lobar hematomas had a greater prevalence of amyloid angiopathy (A) (37.3%) than those with non-lobar topographies. Conversely, those with deep bleeding had a higher frequency of hypertension etiology (H), 82.1%, compared to 9.6% among patients with hematomas in other topographies (p < 0.01).

A comparison of the distribution of the modified Rankin Scale (mRS) scores 10 between etiological subgroups revealed a statistically significant difference, with a higher prevalence of mRS scores of 0-2 among patients with structural injuries (68.8%) compared to those with etiologies associated with the use of medications (10%; p = 0.01).

## DISCUSSION

Our study was the first to evaluate the SMASH-U etiological classification in patients with intracerebral hemorrhage (ICH) in a Brazilian series. We found that hypertensive etiology and structural causes (S1) were more frequent, while amyloid angiopathy (A) and ICH due to medication use were less frequent than previously reported in the international literature. Classifying and studying the etiologies of ICH is of great importance for advancing research and therapies in the area. Developing and applying a unified etiological scale for ICH would facilitate and integrate databases, allowing all researchers to use the same scientific language and facilitating further studies on this topic. Additionally, studying etiology could lead to the development of new therapies. The SMASH-U scale was developed to make the etiological classification of ICH easier and faster, allowing the attending physician to identify the etiology of the event with few additional methods. Its flowchart also allows for identifying the most probable cause (responsible cause) even when the patient has several risk factors and synergistic factors.

In our study, the agreement between evaluators of the SMASH-U scale was found to be good (77.9%). This result is in line with other studies which suggest that the scale presents reproducible results when used by independent evaluators. 14 Such findings indicate that this scale can be used daily with similar results by trained evaluators, thus facilitating the organization and retrieval of patient data and its utilization in future research. Intracerebral hemorrhage (ICH) is a major cause of morbidity and mortality in Brazil and worldwide, accounting for 20% of stroke cases and being the most severe subtype. 5 6 8 14 In our series, ICH represented only 6% of the strokes screened in our outpatient clinic, potentially indicating a selection bias since very severe patients or those who passed away were not evaluated in the outpatient clinic. Out of the 140 patients studied, 20.7% were aged less than 45 years old, and in this population, the etiological classification was found to be different from that of older patients, which may have therapeutic implications. The main cause of ICH in young patients was structural vascular lesions, followed by hypertension and systemic changes. 8 As anticipated, no individuals under the age of 45 were classified as having amyloid angiopathy. Medication use was not found to be an etiology of ICH in patients of this age group. Acquiring a deeper understanding of the etiology of ICH could facilitate the development of public health policies that focus on primary and primordial prevention, especially among younger populations.

We observed a discrepancy in the SMASH-U classification between sexes. Among men, hypertension (52.68%) and amyloid angiopathy (18.4%) were the two primary etiologies, making up 71.08% of patients. Conversely, hypertension (28.12%) and amyloid (18.7%) accounted for only 46.82% of the cases among women. This variation may be partially explained by hormonal protection from estrogen in women of a younger age group and socio-cultural factors, given that women are more likely to access health services for obstetric and gynecological care.

The significant presence of lobar bleeding in our series, even in hypertensive patients, suggests that an association of etiologies can happen. We can still point out a possible etiology in such patients using the SMASH-U classification. Furthermore, deep, infratentorial, and intraventricular bleeding were seen less often than expected, which could result from selection bias, as most patients were recruited from our outpatient clinic.

In our study, the clinical and epidemiological characteristics of the patients evaluated were comparable to those of other national and international series. The most prevalent risk factor observed in our study was SAH, followed by smoking and a history of alcohol use. 7 Surprisingly, few of the patients were taking medications used to prevent cardiovascular disease, such as aspirin (25.2%) and statins (34%). This may indicate the difficulty in accessing primary care services and the challenge of implementing health promotion and quality of life programs. In our series, 9.3% of the patients were using oral anticoagulation, specifically vitamin K and antagonists. Due to the lack of availability of DOACs in the Brazilian public health system, this result is expected. Notably, out of the thirteen warfarin patients, three were not classified as having a drug-induced etiology according to the SMASH-U criteria.

Patients with structurally induced ICH (S1) had a higher likelihood of achieving functional independence, while those with etiological ICH caused by medication use (M) had a lower likelihood. These results are consistent with those previously reported in the original description of the SMASH-U scale. 4

Our study had some limitations. As it was a single-center retrospective series, data were collected from medical records, some of which were not available including blood pressure levels. We included patients who had been admitted to our hospital or visited our outpatient clinic during the study period; however, patients evaluated in the emergency room or who died within the first 24 hours were not included, potentially introducing a selection bias towards less severe cases. Finally, our study, while introducing and describing the classification within a Brazilian population, did not undertake an assessment of its external and internal validity.

In summary, our study elucidates the etiological classification of ICH patients in a specialized Brazilian stroke care center, with implications beyond the national context. Hypertension and amyloid angiopathy were the most frequent etiologies identified. In younger patients, structural injuries and systemic diseases were more prominent. The clinical and epidemiological characteristics of the patients studied were like those of other national and international series. Similar investigations across diverse Brazilian centers could further enhance our comprehensive understanding of this matter.
